# Vaccines in Breast Cancer: Challenges and Breakthroughs

**DOI:** 10.3390/diagnostics13132175

**Published:** 2023-06-26

**Authors:** Gul Naz Fatima, Hera Fatma, Shailendra K. Saraf

**Affiliations:** Division of Pharmaceutical Chemistry, Faculty of Pharmacy, Babu Banarasi Das Northern India Institute of Technology, Lucknow 226028, Uttar Pradesh, India

**Keywords:** breast cancer, diagnostics, biomarkers, therapeutics, vaccine strategies, antigens, adjuvants, challenges, patents

## Abstract

Breast cancer is a problem for women’s health globally. Early detection techniques come in a variety of forms ranging from local to systemic and from non-invasive to invasive. The treatment of cancer has always been challenging despite the availability of a wide range of therapeutics. This is either due to the variable behaviour and heterogeneity of the proliferating cells and/or the individual’s response towards the treatment applied. However, advancements in cancer biology and scientific technology have changed the course of the cancer treatment approach. This current review briefly encompasses the diagnostics, the latest and most recent breakthrough strategies and challenges, and the limitations in fighting breast cancer, emphasising the development of breast cancer vaccines. It also includes the filed/granted patents referring to the same aspects.

## 1. Introduction

Cancer is a heterogeneous disease [1,2,3,4,5,6] with a poor median survival rate [3]. According to the WHO, it is the second-leading cause of death worldwide [5,6], with breast cancer (BC) being the most common form diagnosed in females [7]. About 5–10% of patients diagnosed with BC exhibit its metastatic form [8]. Moreover, it is highly challenging to forecast the prognosis of the illness with high certainty [3]. BC is classified as invasive or non-invasive [9,10]. The invasive form includes infiltrating ductal carcinoma (IDC) and invasive lobular carcinoma (ILC), while the non-invasive form includes ductal carcinoma in situ (DCIS) and lobular carcinoma in situ (LCIS) [10]. BC is further categorised depending upon the expressing hormone receptor such as the estrogen receptor (ER+), the human epidermal growth receptor 2 (HER2+), the progesterone receptor (PR+) and triple-negative breast cancer (TNBC), i.e., ER, PR, and HER2-all negative [11]. TNBC makes up 10–30% instances of BC [12] and is distinguished by a higher rate of relapse, higher potential for metastasis, and a shorter overall survival [13]. Additionally, cases of male BC, which may be either congenital, developmental, or acquired [14], have recently increased by about 40%, outpacing female cases by 25% of the affected population and by 18% in terms of mortality [15,16], though accounting for fewer than 1% of total BC diagnoses [14,17,18,19].

The article discusses at length the principle, approaches, and types of vaccines; and the routes of administration, antigens, and adjuvants used in the development of a BC vaccine, including combination therapeutic approaches and the clinical trials taking place in the field, along with a brief discussion on the diagnostics and the treatment strategies.

## 2. Diagnostics and Treatments in BC

In the recent past, various diagnostics have been developed for the detection of cancer in human subjects. The review briefly presents the diagnosis and its advancements, especially in BC detection. These include computer aided diagnosis (CAD), magnetic resonance imaging (MRI), tomography, Raman imaging, mammography, biopsy, radiomics, pathomics, and the use of artificial intelligence (AI), exosomes, and biomarkers.

CAD is a technique that is helpful in detecting cancer in tissue samples and distinguishes healthy from unhealthy tissue [20]. It also classifies cancer stages [21] using artificial intelligence. A digital multiple classifier database, called the Mammographic Image Analysis Society (MIAS), is a recent advancement for the better classification of lesions in mammograms [22,23]. MRI is a potential technique that is helpful in determining, with fineness and accuracy, the size and vascularisation of a tumour tissue [24]. It also provides the best soft tissue resolution [25] and, thus, has become a widely used tool for the in vivo characterisation of BC, which reduces the need for unnecessary biopsies [26]. Related to the tomographic technique, computed tomography (CT) in combination with positron emission tomography (PET) has become more crucial in the staging and restaging of BC [27]. The detection of distant metastases, using 18F-fluorodeoxyglucose positron-emission tomography (FDGePET/CT), was recently reported to have good sensitivity and specificity [28]. Along with MRI, a CT scan is more frequently utilised and offers a greater imaging resolution [29], with a high accuracy of up to 98% [27]. Raman spectroscopy is a quick and non-destructive optical technique [30] that does not require any staining process [31,32] for the diagnosis of human cancer [33]. The major advantage it offers is a label-free way of evaluating biological samples with great molecular specificity [34]. Despite the advanced diagnostics, conventional mammography still plays an important role in detecting BC. It is the art of capturing a picture of the breast [35] using a low-dose X-ray [20,36,37,38,39]. A combination of mammography with MRI or ultrasonography further improves the chances for the early detection of BC [40]. The technological advancement of mammography resulted in the following two forms: contrast-enhanced mammography [41] and contrast-enhanced spectral mammography. These techniques help to improve the sensitivity towards providing the detailed functional particulars of the anatomic and morphologic characters, as compared to a conventional mammogram [42,43]. When a mammogram or other imaging modality identifies some kind of abnormality, it is most likely that the technique called biopsy works. This includes surgical, core, and fine-needle aspiration [20]. Image-guided biopsy, through the utilisation of advanced interventional radiology techniques, improved the accuracy and safety of the procedure [44]. Another technique used is radiomics, which involves the extraction of information from the images obtained from high-dimensional imaging biomarkers. The technique helps to study the mechanisms occurring at the genetic and molecular levels for predictive and prognostic modeling [45,46]. It also enables the quantitative measurement of intra- and intertumoural heterogeneity, thereby enabling analysis of the entire tumour volume [47]. Similar to radiomics but based on a machine learning program, pathomics is useful for the study of breast oncology. It largely employs in vivo/ex vivo imaging, and analysis of the digital microscopy images of tissue, cells, and subcellular structures [46]. An added advantage is that it quantitatively assesses the structural features at multiple magnifications to complement the traditional histopathologic evaluation, with improved prediction of biological behaviour and clinical outcomes to guide treatment strategies [48].

In addition to the above diagnostic methodologies, the latest advancement in recent years is the use of machine learning programs. This has gained enormous popularity in the field of clinical BC research [3]. With its exceptional precision, AI is increasingly being suggested for use in BC screening [49], diagnosis, and prognosis [3,50]. The use of AI in BC risk assessment and prediction is also possible [16]. This uses digital mammography and digital breast tomosynthesis for detection [50,51,52]. Tomosynthesis involves removing the top layers of breast tissue for the ease of visualisation of the features and boundaries of the lesions [41]. AI is expected to be widely used in this field in the near future [52].

Exosomes are membrane-bound extracellular nanovesicles of endocytic origin [53,54,55]. They are present in diverse body parts [56,57] and may be extracted from extracellular fluids [55]. They play a crucial role in the progression of BC and are involved in the stimulation of tumour angiogenesis, the reorganisation of the stroma, and the promotion of tumour growth and create the tumour microenvironment (TME) [58]. Studies showed that exosome contents reassemble with cancer [54], thereby offering hope for lowering malignant cell activity and enhancing cancer imaging, prognosis [55], and liquid biopsy [53]. In addition, the expression pattern of exosomal microRNAs (miRNAs) correlates with the degree of tumour malignancy and prognosis. Therefore, circulating exosome-encapsulated miRNAs present an early prognostic biomarker for BC [58]. Any quantitative signs that reveal the presence or potential of malignancy or forecast tumour behaviour or prognosis are referred to as biomarkers [59]. Studies showed that the presence and the number of axillary node metastases are the most significant prognostic biomarkers in cases of BC. The ER marker is one of the BC prognostic biomarkers that is now available [60]. Currently, the multiple reaction monitoring (MRM) assay has been evolved to detect BC biomarker peptides in serum samples [61]. miRNAs and exosomes are new diagnostic and therapeutic biomarkers for BC patients [57,59]. Some biomarkers for BCB (breast cancer bone) metastases include CST1/2/4/6, PLAU, PLAT, COL6A1, and PLOD2. The advancement of technology, such as deep sequencing, lent advantages over conventional biomarkers by being non-invasive [59]. Proteins such as ER, ERR, Her2, Ki67, CEA, and TSGF and miRNAs such as miR-10b, miR-21, miR-145, and miR-155 are examples of distinct BC biomarkers [57].

The treatment of cancer has always been a challenge for the scientific community. The following paragraphs briefly describe some of the strategies developed over time to treat BC.

One of the most advanced strategies for the treatment of BC involves the use of nanoparticles (NPs). They are an efficient means to deliver medications targeting malignant cells [62], with improved pharmacokinetics and pharmacodynamics [63] and reduced side effects [64]. For example, a liposomal nanoformulation of Doxorubicin was shown to display action with significantly reduced side effects. Catechols, gallol, and their derivatives are used to construct nanomaterials [65], with the use of polymers, to deliver conjugated or encapsulated drugs [66]. Gold, silver, and iron oxide NPs are thermally stable, low in toxicity, compact, and effective in BC treatment [11]. In addition, magnetic NPs are also being studied as promising materials for prospective nanopharmaceuticals [67]. Neoadjuvant treatment is an effective treatment strategy for inoperable locally advanced BC [68]. In patients with non-metastatic BC and axilla tumours, neoadjuvant therapy is used to de-escalate the extent of surgery [69], thereby minimising unnecessary surgeries and mastectomies [70]. In order to fix, add, or repress a gene, the genetic material must be first administered through a vector to the target cells. Gene therapy is less toxic than conventional medicine and helps to target tumour cells without harming normal cells [71].

Radiotherapy (RT) exerts local action [72] and represents one of the most effective non-surgical treatment modalities [73]. Whole breast irradiation, also known as WBI, is frequently carried out following a breast-conserving surgery [29], i.e., intraoperative radiation therapy (IORT), which is a therapeutic strategy that accomplishes surgery and adjuvant RT in a single treatment [29].

The dynamic relationship between tumour cells and the immune system involves three key stages: elimination, equilibrium, and escape [74]. Immunotherapy (IT) is a strategy that works by stimulating and recruiting a patient’s immune system against the tumour cells [75,76,77]. Various IT approaches include tumour-targeting antibodies, adoptive T-cell therapy, vaccines, and immune checkpoints [60]. Glycoprotein mucin-1 (MUC-1) was one of the first BC-associated antigens to be identified [78]. In order to evaluate the cell–cell interactions, the use of bioengineering tools such as optical microscopy and a microphysiological in vitro system was accomplished [79]. IT, however, can negatively affect the foetus or impede a future desired pregnancy [80].

The use of combination therapy may give a new hope for BC treatment [81,82]. It is a promising therapeutic approach that involves folate ligands in conjunction with chemotherapeutic drugs and small interfering RNAs (siRNAs) [83]. A study undertaken by Ma et al. reported the beneficial effects of a combination of Trastuzumab and Everolimus in patients with ER+/HER2-HER mutant BC [84]. It reported a boost in the survival rates of patients by up to 35%. Some examples of combination approaches include the combinations of any of the following therapies: immunotherapy, molecular-targeted therapy, cell cycle management, cell signalling pathway regulation, monoclonal antibodies, and antibody–drug conjugates [60].

Thermal ablation (TA) therapy exerts its action by destroying the entire tumour using heat, through needle-like applicators [85]. It results in minimal invasion without damaging the adjacent vital structures [86,87] and, thus, is a safe and effective alternative to surgical resection in early-stage treatment of BC [88]. This advancement resulted in percutaneous TA techniques, viz., radiofrequency ablation, microwave ablation, cryotherapy [89], and cryoablation [90,91]. Another method is radiofrequency ablation (RFA), which entails inserting a metal electrode into the breast and connecting it to a radiofrequency generator that outputs 200 W at 400–800 KHz. [91]. The tumour tissue is burned at temperatures (50 °C) high enough to cause necrosis [92]. Microwave (MW) ablation is a recent advancement in the field that involves heating malignant tissue with MW energy to cause cell death [91]. The goal of hyperthermic therapy is to kill tumour cells by inducing a series of metabolic changes, such as apoptosis in the tumour tissue by heating it to high temperatures [93]. The use of magnetic nanoparticles (MNPs) such as iron oxide nanoparticles (IONPs), in an alternating magnetic field results in magnetic hyperthermia. The technique utilises two types of magnetic nanoparticles: magnetic alloy nanoparticles (MANPs) and magnetic metal oxide nanoparticles (MMONPs) [94]. They are site-specific and are classified as whole-body hyperthermia, regional hyperthermia, and localised hyperthermia depending on the site of application [95].

In addition, modern medicine emphasises the prevention and treatment of cancer with natural nutritional components [96]. This includes the use of flavonoids, phenolic compounds, and some dietary sources that effectively exhibit anticancer activity [96,97,98,99]. For example, quercetin prevents free radicals from impairing low-density lipoproteins and is useful against cancer [96]. Luteolin [99] and resveratrol proved to modulate various signaling pathways involved in the proliferation of cancerous cells and are established as anticancer agents [100]. Some naturally occurring constituents such as the *ϖ*-3 fatty acids and phytoalexin found in grapes; epigallocatechin gallate, a polyphenolic compound found in herbs such as *Oldenlandia diffusa* and *Ziziphus jujube* [98]; and Silibinin, a major component of Silymarin that is obtained from the seeds of milk thistle, *Silybum marianum* [97], can be used in the treatment of BC. Pomegranate, *Punica granatum*, can also be used against different type of cancers [101].

## 3. Vaccine Therapy in BC

### 3.1. Introduction

Vaccines have long protected humans from communicable and non-communicable diseases [102]. Therefore, conventionally, the word “vaccine” is usually related to the fight against infectious diseases. Vaccines exert their action by stimulating immune responses. This is achieved by inoculating a healthy individual with attenuated/detoxified bacteria, viruses, or extracted toxins [103].

The immune system works to keep living things in a state of equilibrium by the process called immune surveillance [104]. The method by which tumour cells circumvent the immune system has been extensively explored and has been successfully established over the past few years. Such efforts led to the conclusion that *cancer immunoediting* is the strategy employed by the tumour cells towards immune evasion [105,106]. It may be caused by TME-antigen-mediated antitumour immunological responses. A number of tools for cancer immunotherapy were developed, which include antibodies, peptides, proteins, nucleic acids, and immune-competent cells such as dendritic cells and T-cells [103]. Cancer immunotherapies are now considered the fourth treatment method [107,108], used either alone or in combination [60]. The therapy was initially utilised by William B. Coley in 1891, to treat sarcoma patients with Coley’s toxin [109].

The available vaccines for cancer immunotherapy can be divided into two basic types, the prophylactic and the therapeutic vaccines [110,111]. The former induces immunological memory by vaccinating healthy people [112], to prevent morbidity from a certain malignancy [113], and can be a cost-effective preventive measure [114]. The latter boosts immune systems in people detected with cancer [103]. The therapeutic cancer vaccine prevents the growth of advanced malignancies or relapsed tumours that are resistant to standard treatments [112]. Based on the structure and the content, the vaccines are further classified into the cell vaccines, the peptide vaccines and the nucleic acid vaccines [103].

The criteria necessary to be fulfilled to achieve vaccination requires a target antigen on tumour cells to stimulate the immune response, a vector to deliver the vaccine-derived antigen to the immune system, an adjuvant to boost immunological stimulation, and an appropriate monitoring tool [115]. These are discussed in detail in the subsequent sections.

### 3.2. Concepts in Designing of a Breast Cancer Vaccine (BCV)

#### 3.2.1. Immunoediting

**Figure 1:**
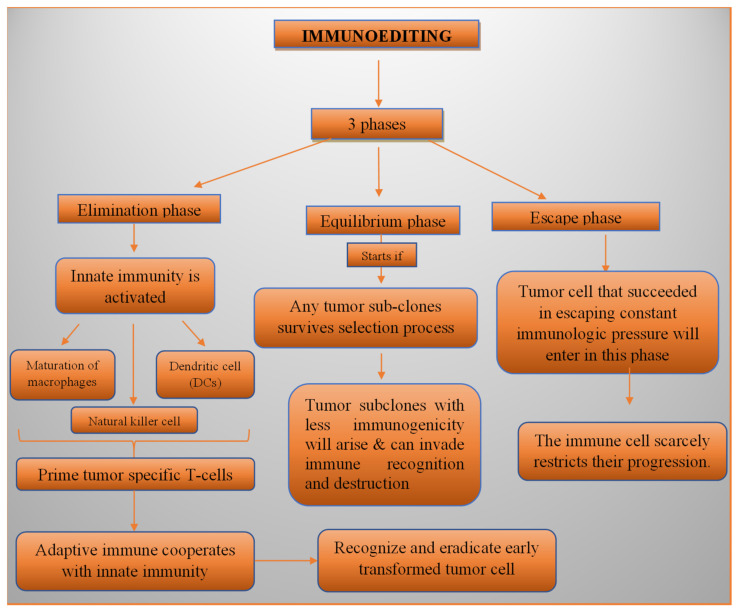
The process of immunoediting carried out by cancer cells.

The immune system constantly changes as BC progresses. The process is called immunoediting, which comprises three steps: elimination, equilibrium, and escape. During elimination, the tumour cells stimulate the innate immune system (which along with the adaptive immune response can recognise and remove early altered tumour cells) through the activation of macrophages, natural killer cells (NK-cells), and dendritic cells (DCs), in turn activating the tumour-targeted T-lymphocytes. The equilibrium phase initiates in the case of a cancer subclone colony surviving the host’s immunity. This stage creates a delicate balance between cancer growth and the immune system’s defence function, making it difficult to totally remove the tumour cells, though their progression is severely limited [116]. However, it results in the formation of cancer cells with decreased immunogenicity, epigenetic alterations, and genetic instability [117], thus making them capable of escaping the immune detection and destruction [118]. Such cells that escape the immunological pressure finally enter the third stage of immunoediting, where the immune system barely puts any restraints on the progression of the modified tumour cells as in Figure 1 [119].

#### 3.2.2. Immune Surveillance

Effector immune cells must directly recognise tumour antigens from tumour cells or indirectly from antigen-presenting cells (APCs), via the major histocompatibility complex (MHC) on the cell surface, to initiate an immune response. CD8+ and CD4+ T-cells are critical to the immunoediting process and help separate the non-self epitopes of the tumour cells expressed by MHC class I and MHC class II molecules from the normal self-antigens [120,121]. Tumour-specific antigens (TSAs) and tumour-associated antigens (TAAs) [122] are examples of tumour antigens. These include germ-line antigens, tissue differentiation antigens, and overexpressed antigens, exemplified by the melanoma-associated antigen, the carcino-embryonic antigen (CEA), and HER2 and MUC-1, respectively [123]. Many of the tumour antigens employed in immunotherapy are expressed in normal tissues as well. However, tumour cells overexpress these antigens [122].

#### 3.2.3. Immune Suppression

**Figure 2:**
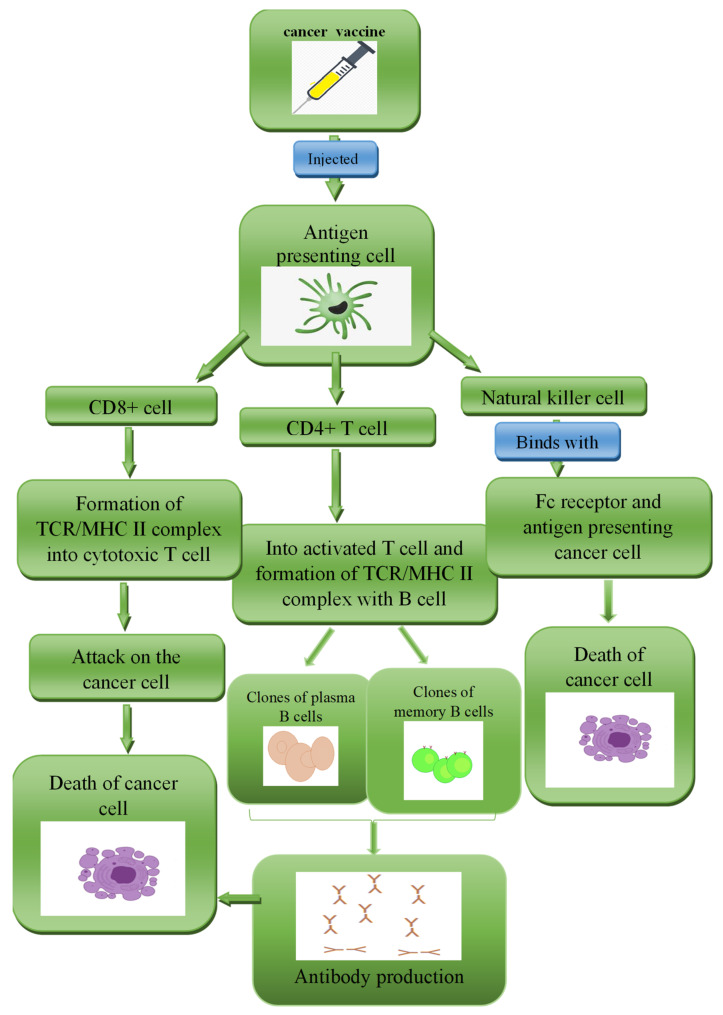
Immune-suppression by tumour cells.

Tumour cells successfully inhibit the host’s immune system, both locally and systemically, from evading immune surveillance [124]. It is well-established that there is predominance of the immunosuppressive effect with advancement of the disease. This results in a gradual transition from the elimination phase to the escape phase [125]. During this process, there is an alteration of the number of cells around TME and the lymph nodes in the vicinity of the tumour tissue. First are regulatory T-cells (Treg cells). There is enhancement in the proliferation of Treg cells brought about by transforming growth factor-*β* (TGF-*β*) [126], while cytotoxic T-lymphocytes (CTLs) are greatly reduced, as interleukin-2 preferably binds to Treg cells. The antitumour response is, thus, weakened by a reduction in the number of CTLs [127] and NK-cells [128] in TME (Figure 2). Treg cells function to downregulate the dendritic cell co-stimulatory markers, viz., CD80 and CD86, necessary for the priming of CTLs [129], which, in turn, function to eliminate any abnormal-phenotype-expressing cell. Second are tumour-associated macrophages (TAMs). TAMs release inhibitory cytokines including IL-10 and TGF-*β* to suppress CTL activity and the production of IL-12 [130]. Third are the myeloid-derived suppressor cells (MDSCs). During the shift, these cells start appearing in the peripheral blood as well [131]. Tumour cells also activate immunological checkpoint receptors such as cytotoxic T-lymphocyte antigen-4 (CTLA-4) and programmed cell death receptor-1 (PCDR-1) [132]. PCDR-1 blocks programmed cell death ligand-1 (PCDL-1).

Therefore, in patients with diverse malignant tumours, the tumour-infiltrating lymphocytes (TILs) and the tumour-specific T-lymphocytes show high amounts of PCDR-1. The involvement of PCDL-1/PCDR-1 by tumour cells prevents the elimination of T-cells, thereby causing their dysfunction and, ultimately, cell death. In contrast, the cytokines that create an immunosuppressive milieu help to further promote tumour growth [133].

#### 3.2.4. Identification of the Antigen for BC Immunotherapy

Numerous tumour antigens, which are expressed in healthy cells but are overexpressed in tumour cells, are employed in BC immunotherapy. They include *HER2*, *p53* (tumour protein 53), *MUC1*, carcinoembryonic antigen (*CEA*), telomerase reverse transcriptase (*hTERT*), and carbohydrate antigens [78]. Due to their widespread expression in the majority of tumour types, some of these antigens are known as universal tumour antigens. An example of this class is *hTERT* [134]. All the potential antigens that are used in the creation of vaccines for the management of BC are briefly discussed in the following section.

##### Human Epidermal Growth (HER2) Receptor 2

*HER2* is a tyrosine kinase that regulates cell proliferation and survival. The oncogene for *HER2* is located on chromosome number 17q12. In cases where BC is *HER2*-positive, an amplification of 15–20% in the expression of *HER2* is found. This is responsible for triggering the growth and progression of tumour cells [135]. *HER2* is linked to a more severe form, a higher risk of recurrence, and a higher mortality rate. The first drug to be established as a *HER2* blocker was Trastuzumab [136], which has been validated for the treatment of patients with *HER2+* BC [137,138].

##### Tumour Suppressor p53 Protein (p53)

The gene encoding *p53* is located on chromosome number 17 [139]. It plays a vital role in the maintenance of DNA integrity and the prevention of cancer. Under normal physiological conditions, when a cell’s DNA is damaged, there is an induction of the *p53* protein that causes arrest of the cell cycle. This allows cells to repair themselves, but, if the damage is too severe, the cells apoptise and are rejected. In a variety of cancer forms, mutations in the *p53* gene (*mtp53*) takes place [140]. However, *mtp53* is less frequently found in BC [141], though it has high significance in the diagnosis and prognosis of TNBC, with almost 70%–80% of cases displaying *mtp53* [142].

##### Mucin1 (MUC1)

The *MUC1* gene is encoded by chromosome 1q21. It is a high molecular weight transmembrane glycoprotein that functions as a physical barrier to protect the epithelial layer of cells from environmental exposure. It forms the epithelial lining of the respiratory and GI tracts, mammary glands, pancreas, liver, and kidneys. *MUC1* is a poly-morphic type I member of the mucin family. It is overexpressed in about 90% of human BC due to genetic alterations and the dysregulation of transcription [143,144]. *MUC1* is also aberrantly glycosylated [145], which exposes various antigens, thereby generating a new set of antibodies that can prove beneficial in the diagnosis of cancer [146].

##### Carcinoembryonic Antigen (CEA)

The CEA is a glycoprotein that is encoded by human chromosome 19q13.2. This antigen is an important serum biomarker for the detection of cancer and plays a vital role in the prognosis and diagnosis of BC. It is found elevated in patients with metastatic BC, especially with bone metastasis [147,148]. Several studies suggested the significance of CEA and CA15-3 levels in predicting BC patients who can be operated on at an early stage [149].

##### Human Telomerase Reverse Transcriptase (*h-TERT*)

The gene encoding *h-TERT*, a catalytic subunit of the telomerase, is encoded on chromosome 5p15.33. This ribonucleoproteic enzyme is responsible for synthesising telomeres, which, in turn, maintain the chromosomal length. The ultimate result is cellular immortalisation [150]. Studies suggested their vital role in the development of cancer. It was found that in most forms of cancer, the reactivation of telomerase takes place in a dependent or an independent manner. The enzyme ensures the stability of chromosomes, thus bypassing senescence. *TERTp* is the promoter region of *TERT*. Mutations in the *TERT* gene support carcinogenesis with variable frequencies, which takes place through the healing of the telomere’s length, thus expanding the life of cells. *TERTp* was established as a tool to characterizes the type of cancer [151] and, thus, is helpful in the diagnosis and prognosis of the disease [152].

### 3.3. Design Approaches of a BCV

The optimisation of vaccine schedules and administration methods is a component of a vaccination strategy. Based upon the platforms and formulations, BCVs can be broadly classified as peptide- and protein-based [153], carbohydrate antigen-based, whole-cell-based, gene-based [154], and fusion-based vaccines [155]. However, irrespective of the type, all vaccines are dependent on the autologous immune system recognising a specific antigen to exert a therapeutic effect. Moreover, the use of an adjuvant is essential, as it helps to increase the antigen immunogenicity, thereby controlling the immune response [156].

#### 3.3.1. Peptide- and Protein-Based Vaccine (PV)

**Figure 3:**
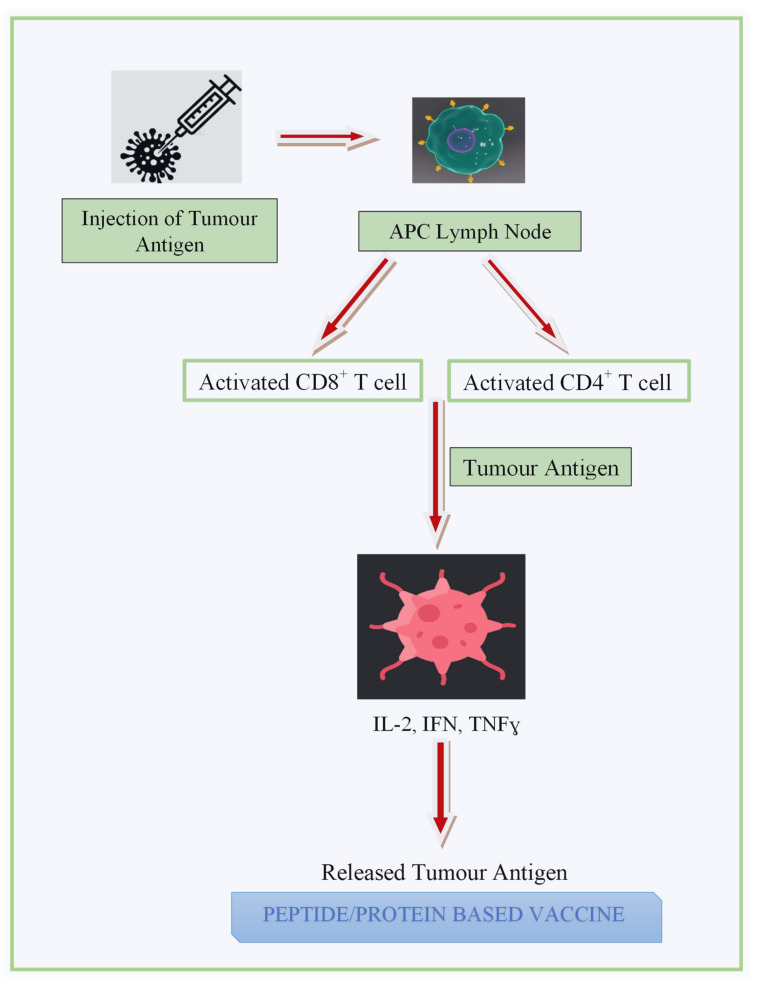
Mechanism of a peptide-based BCV.

In this class of BCVs, the MHC class I restricted peptide epitopes are used to stimulate the immune response against a tumour antigen as shown in Figure 3 [157]. The injected peptide stimulates immune effector cells to find and kill cancer cells [158]. Some short amino acid peptides are preferably used, as they are cheap, stable, and easy to synthesise and modify and display low immunogenicity [159]. However, a vaccination cannot be given to patients with a non-common human leukocyte antigen type, since each peptide is confined to a specific HLA subtype.

MHC class I binding peptides poorly stimulate CD4+ helper T-cells and also, in turn, limit their ability to activate CD8+ cytotoxic T-cells, resulting in a transitory immunological response. According to Pallerla et al., long peptides are capable of containing many MHC class I and class II epitopes, thus helping to partially overcome this difficulty. Such peptides containing 23–45 amino acids may improve T-cell activation through processing and presentation [154]. The success of the peptide-based BCV can be seen from its entry into phase I/II of clinical trials [160].

The entire tumour antigen protein or a truncated portion of it, wherein the sequence of amino acids is substantially longer than that of peptides, is used to create protein-based vaccines [161]. It is not HLA-restricted and allows for the absorption, processing, and presentation of a variety of MHC class I and class II peptide epitopes [162]. However, the presenting method may be less effective, and the lack of a precise marker makes it difficult to estimate how well such vaccine types would work [163]. A number of clinical trials (Table 1) are currently in the pipeline to test the efficacy of vaccines pertaining to BC [162].

#### 3.3.2. Carbohydrate Antigen-Based Vaccine (CAV)

Immune cells can discriminate between improperly expressed carbohydrate antigens in tumour cells. This makes the carbohydrate antigen a prime candidate for inclusion in cancer vaccination. An example includes the expression of a unique disaccharide carbohydrate, Sialy-Tn (STn), on the cell surface of cancer cells, including BC cells, which is connected to MUC-1 [164]. According to Munkley (2016), immunisation with STn results in tumour regression and prolongation of survival time. Thus, it could be beneficial in the development of a cancer vaccine [165].

#### 3.3.3. Whole Tumour Cell-Based Vaccine (WTCV)

One of the conventional approaches in the development of a cancer vaccine is the stimulation of the immune response by the use of whole tumour cells or the products obtained from tumour cell lysis. A WTCV induces a polyvalent immune response, since it is based on a pool of unknown antigens created by autologous or allogeneic tumour cells [166]. Sometimes, the enhancement of the antigen-presenting ability of the WTCV is achieved through engineered tumour cells that are capable of releasing cytokines or expressing co-stimulatory molecules. The drawback of the WTCV is that it contains endogenous cellular antigens, which can result in an autoimmune response. A systematic procedure for creating the WTCV is, however, lacking [167].

#### 3.3.4. Dendritic Cell-Based Vaccine (DCV)

Upon migration into the lymph nodes, a diverse population of APCs, called DCs, effectively absorb antigens to process and present them to CD4+ and CD8+ T-cells. DCs can also stimulate NK-cells and B-cells. DC-based vaccines typically use ex vivo generated DCs that were transfected to express tumour antigens or are loaded with tumour antigens. In a review by Butterfield et al., the use of antigens, such as complex tumour lysates and several MHC class I and class II peptides, and monocytes and CD34+ progenitor cells for the purpose of DCV preparation was discussed [57]. The technical challenge that arises while developing DC-based vaccines was because of the distinct process of ex vivo DC maturation [168].

#### 3.3.5. Gene-Based Vaccine

The strategy for a gene-based vaccine involves a plasmid that carries the DNA encoding the cancer antigen. This form of vaccine can be used to activate both a non-specific innate immunity and an adoptive immunity specific to an antigen. Due to its simplicity, safety, and cost-effectiveness, this approach is regarded as one of the most practicable methods for cancer immunotherapy. However, it suffers from limitations such as a lack of plasmid uptake and ineffective antigen expression, thus presenting insufficient immunogenicity [162]. To overcome this demerit, various approaches were undertaken. One of the approaches was to make the vaccine self-replicating. This utilised the RNA replicase encoding gene [169]. RNA-based medications have the potential to be effective pharmacological regulators against cancer cells by altering the expression of particular proteins. These characteristics help to increase specificity and reduce the chance of off-target impacts [153].

#### 3.3.6. Fusion Vaccine

This strategy employs the fusion of autologous DCs and autologous whole tumour cells. It involves the cytoplasm of both cell types to be fused together without their nuclei doing so. This preserves their ability to function as individual cells. With the fusion, the formed vaccine can express and process a wide range of recognised and unrecognised tumour antigens [170]. Studies and trials found a favourable response by patients with metastatic BC towards this fusion vaccine, in terms of its potent antitumour effects [154,171].

### 3.4. Adjuvants Used in Design of BCVS

Adjuvants are combined with the antigen and are essential in the case of low immunogenicity. They help to increase the immunogenicity of the antigen and thus, trigger, the immune response, particularly in the elderly [156]. Most adjuvants work by decreasing antigen release, encouraging antigen absorption and presentation by APCs, and boosting the growth of DCs and macrophages [172]. Traditional adjuvants, such as alum, mostly stimulate type-2 T-helper cell-dependent humoral immunity in prophylactic vaccinations for infectious diseases rather than type-1 T-helper cell responses that directly destroy tumour cells [173]. A common adjuvant in BCV is a secreted cytokine, called granulocyte-macrophage colony-stimulating factor (GM-CSF). It was demonstrated to promote the proliferation and activation of DCs as well as the maturation of myeloid cells such as granulocytes and macrophages [174,175]. Clinical trials of many GM-CSF-containing BCVs revealed measurable immune responses. The local administration of GM-CSF to melanoma patients increases the probability of the antigen immune response after vaccination. Studies found GM-CSF suppresses T-cell responses and produces inhibitory MDSCs. However, elaborative research is needed to discover the role of GM-CSF as an adjuvant for cancer vaccines.

**Figure 4:**
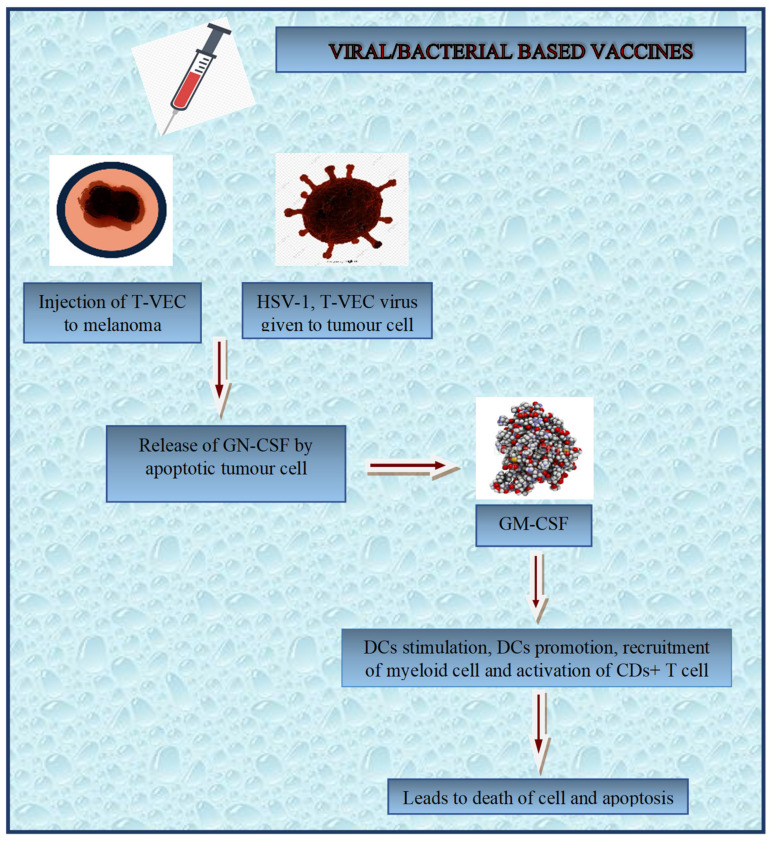
Diagrammatic representation of vector-based vaccines.

DNA-based cancer vaccines also use recombinant viral vector adjuvants (Figure 4). Recombinant viral vectors, which commonly carry antigens, contain different levels of pattern recognition receptor (PRR) and toll-like receptor (TLR) ligands that activate DCs and boost immune response. TLR agonists activate CD8+ T-cells and prevent T-cell exhaustion. The vectors contain other sequences that can compete with the targeted antigen motif, which is the main drawback of this adjuvant. However, adjuvant effects vary depending on vaccination formulation, targeted tumour antigens, immunisation schedule, and mode of administration, making adjuvant comparisons difficult [176]. Moreover, adjuvant optimisation tests for BCV are crucial.

### 3.5. Routes of BCV Administration

A good vaccine for cancer should be capable of efficiently transferring antigens to autologous APCs. For this purpose, various strategies are preferred. HER2 peptide-based vaccines are usually intradermally administered and exhibit an enhanced rate of response. This is probably due to the widespread network of DCs. Low intradermal peptide doses are safe and trigger specific responses by antigen T-cells in most healthy human subjects [177,178,179]. Several BCVs were tested for their immune response induction efficiency via subcutaneous injection [180]. However, a large dose of antigen at the site of injection may cause severe reaction and sporadic sterile abscesses, which may require vaccine discontinuation or a dosage reduction [175]. Vectors or plasmids are injected through various routes in the case of the administration of a DNA-based vaccine, of which the intramuscular route of administration was found to display the most effective immune response [181]. Some DC-based immunisations must be given intravenously to directly stimulate lymph node T-cells [157].

### 3.6. Clinical Trials of BCVs

In preliminary studies, certain BCVs were successful in eliciting discernible immune responses and showing good tolerance. However, most of them showed appreciable clinical advantages in the ensuing Phase 3 trials. Recently, the NeuVax^TM^ vaccine (developed by Galena Biopharma, a US-based biotechnology company), which is administered with Leukine^®^ for the disease condition of BC with intermediate to low HER2 expression, was granted a Special Protocol Assessment for its Phase 3 trial (Prevention of Recurrence in Early-Stage, Node-Positive Breast Cancer) [182]. The Theratope^®^ and Enhanzyn™ vaccines are some of the vaccines that were withdrawn from the clinical trial enrolment [183]. A brief summary of the vaccines in clinical trials is listed in Table 1.

### 3.7. Combinational Therapy of BCVs

Immune checkpoint blockers (ICB) have changed the approach towards the treatment of cancer. As far as BC is concerned, ICBs were already proven to be effective in treating metastatic TNBC [184]. The addition of ICB to Trastuzumab, however, was linked to additional side events and did not provide a clinically substantial improvement in the progression-free survival for HER2-positive metastatic BC [185]. Combining the vaccination with ICB to combat cancer tolerance is a trending research approach in the field [186]. As previously stated, ICB blocks inhibitory receptors such as PCDR-1/PCDL-1 and CTLA-4 to enable the effector immune cells to kill tumour cells. According to certain preclinical research, when T-cells are activated by tumour vaccines, the inhibitory receptor expression on the cell surface also increases. One of the underlying reasons is the enhanced interferon-*γ* (IFN-*γ*), which is released by tumour-specific T-cells. IFN-*γ* up regulates the expression of PCDL-1 on the tumour cells and APCs. PCDL-1 initially helps to prevent the body’s immune responses from being too amplified [187,188]. Therefore, the immunosuppressive impact that reduces the antitumour immunity elicited by vaccines is likely to be relieved by an injection of ICB [189]. A promising approach that has the potential to improve and lengthen the course of the immune response and effectively produce considerable clinical benefits is the combination of the BC vaccination with ICB. Additionally, combining cancer vaccinations with recognised medicines could also increase efficacy.

The research suggests that some HER2-derived peptide vaccines [190] and anti-HER2 monoclonal antibodies may function synergistically [191]. Studies show a connection between chemotherapy/radiation therapy and immune-related cell death. When these medicines are applied in conjunction with cancer vaccines, it might create a long-lasting immune response. Consistently, it would be worth investigating the effects of integrating cancer vaccination with chemotherapy [192,193], hormone therapy [194,195], targeted therapy [196,197], and radiation therapy [198,199].

### 3.8. Challenges Faced during the Course of Development of BCVs

Despite the several advantages associated with cancer vaccines, there are numerous challenges accompanying cancer vaccination strategies. Firstly, the development of tolerance towards the antigen, which results in lowered immune response. Secondly, the heterogenicity of the tumour type brings in some diverse intrinsic and extrinsic pressures, which, in turn, result in a detrimental effect on the antitumour vaccination processes. In addition, some genetic and non-genetic mechanisms play a role in defining the heterogenicity of cancerous cells, thus affecting the immune response. Moreover, the immune invasion mechanism was found to be a major hurdle in the efficacy of a vaccine. This requires potential alternative strategies to improve antitumour immunity [200]. Some developed vaccines were found to display poor immunogenicity when used alone. Therefore, the next generation of adjuvants may be used [201]. A key point during the development of an anticancer vaccine is the aftermath, in the form of immunological responses. Thus, it is necessary to recognise the immune modulatory pathway, and test and validate the disease-specific cancer vaccination [202]. Cancer immune interaction is also governed by the concomitant use of a drug.

## 4. Recent Patents Filed/Granted on Cancer Treatment and Diagnostics

This review also encompasses the patents filed/granted at the USPTO during 2018–2022, as shown in Table 2 [203,204,205,206,207,208,209,210,211,212,213,214,215,216,217,218,219,220,221,222,223,224,225,226,227,228,229,230,231,232,233,234,235,236,237,238,239,240]. The patents present advancements in diagnostic technologies as well as in cancer therapeutics.

## 5. Conclusions

Breast cancer (BC) is one of the most prevalent diseases worldwide. However, advancements in science and technology have aided the development of methods for BC diagnosis. They have also transformed the way BC is treated. As far as BC therapeutics are concerned, a revolution was introduced by the use of the immune system as an instrument. This led to the development of several promising BCVs, including preventive, therapeutic, and fusion vaccines. Even though many strategies have been employed to date, developing a cancer vaccine still remains challenging. The major challenges faced when creating a cancer vaccine include inadequate immunogenicity, tumour heterogeneity, poor antigen identification, and the health consequences.

An overview of the approaches for diagnosing BC, related treatments, vaccines, and patents was included in the article to provide leads for future research. Thus, with the advancements in research tools and new technologies, cancer vaccines may soon be a reality.

## Figures and Tables

**Table 1:** Clinical trials in the field of breast cancer vaccines.

NCT Number	Antigens/Biological	Clinical Phase
NCT00854789	E75 and GM-CSF	I
NCT00892567	Her-2/neu; CEA and CTA	I
NCT02019524	E39 and J65 peptides	I
NCT04270149	ESR1 peptide vaccine	I
NCT04521764	*Helicobacter pylori* neutrophil-activating protein	I
NCT00343109	HER-2/neu	II
NCT02348320	Personalised polyepitope DNA vaccine	I
NCT02018458	LA TNBC; ER+/HER-BC	I/II
NCT04348747	Anti-HER2/HER3 DC vaccine; Pembrolizumab	II
NCT02061423	HER-2 pulsed DC vaccine	I
NCT01730118	AdHER-2/neu DC vaccine	I
NCT00524277	HER2-derived peptide GP2; GM-CSF	II
NCT01479244	HER2-derived peptide E75; GM-CSF	I/II
NCT01570036	HER2-derived peptide E75; GM-CSF; Trastuzumab	II
NCT00140738	HER; AS 15	I/II
NCT02061332	HER; DC vaccine	II
NCT00399529	HER2; GM-CSF; Cyclophosphamide; Trastuzumab	II
NCT01479244	HER2-derived peptide E75; GM-CSF	III

**Table 2:** Some patents related to cancer diagnosis and treatment filed/granted in the past five years.

S. No.	Patent Number	Date of Publication	Invention Disclosed
1.	US 2022/0195008 A1	23 June 2022	This invention disclosed the antigenic specificity of the T-cell receptor (TCR) for the melanoma antigen family. The polypeptide in the functional portion of the TCR was found to carry amino acid sequences of length 16–21 [203].
2.	US 2022/0193199 A1	23 June 2022	This invention described an immune cytokine that is a conjugate and an immunomodulatory antibody. It comprised an interleukin15-containing polypeptide, which is an IL-15Ra sushi domain-containing polypeptide. The immunomodulatory antibody- or antigen-binding fragment was said to be capable of binding PD-1 and PD-L1/L2 [204].
3.	US 2022/0193079 A1	23 June 2022	A pharmaceutical combination comprising a CDK inhibitor and an antihormonal agent that regulates the P13K/Akt/m TOR pathway or a pharmaceutically acceptable salt was discussed in the patent [205].
4.	US 2022/0170012 A1	22 June 2022	This invention disclosed the compositions and methods of generating an RNA chimeric-antigen receptor of transfected T-cells for use in adoptive therapy for cancer. The method modified the 5′ end of the RNA or its 7-methyl guanosine cap by the addition of the 5′-end of the eukaryotic messenger, soon after the initiation of the transcription [206].
5.	US 2022/0184111 A1	16 June 2022	Methods for reducing the cytotoxicity of chemotherapeutic agents towards non-cancer cells and increasing their cytotoxicity towards cancer cells were described in this patent. The patent gave details regarding the administration of an effective amount of the agent to achieve the inhibition of CD47 signalling for an effective chemotherapeutic agent [207].
6.	US 2022/0185892 A1	16 June 2022	This patent disclosed a method for the treatment of a solid tumour through the administration of an effective quantity of some anti-LAG-3 and anti-PD1 antibodies that carry the CD-R1, -R2, and -R3 domains of the chain [208].
7.	US 2022/0186323 A1	Jun16, 2022	This invention discussed a method for detecting a tumour marker, which, in turn, indicated the presence of a cancer. The marker may be a sample of nucleic acid. The sample was then amplified by polymerase chain reaction, thereby enriching the nucleic acids for the detection of genomic regions [209].
8.	US 11,291,723B2	5 April 2022	A treatment method for enhancing the efficacy of a therapy for cancer in humans and animals was presented in this invention. This treatment was selective in killing or reducing the growth of the target cell by using the enzyme- Cas nuclease. It was also related to cell populations, system, arrays, cells, RNA, and other means affecting the therapy [210].
9.	US 2022/0040278 A1	10 February 2022	This patent disclosed cancer immunotherapy relating to tumour-associated T-cell peptide epitopes. The presence of peptides on the tested tissue sample biopsies helped to diagnose cancer. This patent also gave methods, such as antibody detection or spectrometry, for analysing peptides [211].
10.	US 2022/0010385 A1	13 January 2022	This invention discussed a method to detect the inactivation of the DNA homologous recombination pathway, which helped to detect the BC gene, BRCA, or placental alkaline phosphatase, PALP (also called FANCN), inactivation, which was probably due to a somatic mutation or a mutation in the germ cell line [212].
11.	US 11,220,715B2	11 January 2022	An expression of a set of genes as thera-prognostics for the disease-free survival of cancer patients was disclosed in this invention. It gives the use of a paraffin-embedded biopsy material that is compatible with different methods of tumour tissue harvest [213].
12.	US 2022/0003792 A1	6 January 2022	This invention mentioned the methods to determine the presence or absence of a cancer type in an animal. The procedure determined the concentration of lipid amounts in a sample, which was obtained either from a detected cancer or a treated individual’s body fluid, using the mass spectrometric technique [214].
13.	US 11,085,084B2	10 August 2021	This patent gave details regarding the identification and analysis of some polynucleotide adaptors, which include cell-free nucleic acids, from sample cancer patients. It also included their detection, diagnosis, and prognosis [215].
14.	US 2021/0145835 A1	20 May 2021	A pharmaceutical composition that could be utilised for the treatment of patients diagnosed with HER2-positive BC was addressed. The pharmaceutical substance disclosed was (R)-1-(3-(4-amino-3-(4-phenoxyphenyl)-1H pyrazolo [3,4-d] pyrimidin-1-yl) piperidin-1-yl) prop-2-en-1-one [216].
15.	US 2021/0137936 A1	13 May 2021	This invention described the administration of a therapeutically effective quantity of N-((4,6- dimethyl-2-oxo-1,2-dihydropyridin-3-yl) methyl) -5-(ethyl (tetrahydro-2H-pyran-4-yl) amino)-4- methyl-4′-(morpholino-methyl)-[1,1′-biphenyl]-3-carboxamide hydrobromide. The compound was a polymorph and exhibited an X-ray powder diffraction pattern with characteristic peaks [217].
16.	US 10,991,448B2	27 April 2021	This patent related to methods for the evaluation of the diagnosis of cancer in a patient. The patient may be subjected to some therapy. The attributes that were measured included the degree of mutation, transcription and translation levels, protein, and interaction. Thus, a probabilistic pathway gave a comparative graph representing the cellular processes [218].
17.	US 10,987,037 B2	27 April 2021	This invention was related to the collection of live tumour cells using a collecting probe, where the procedure involved placing the probe in a living organism. The probe used was composed of guide wire carrying a binding surface with an optically sensitive dye and an atraumatic tip at the distal end [219].
18.	US 2021/0093715 A1	1 April 2021	A method for the treatment of patients with metastatic BC that would extend the progression-free survival of the subjects under treatment was disclosed. The therapeutic regime consisted of an effective dose of a chemotherapeutic agent and an anti-VEGF antibody [220].
19.	US 2021/0047429 A1	18 February 2021	This invention disclosed a method for the treatment of BC with an effective amount of HER2 antibody and a taxane derivative [221].
20.	US 2021/0040216 A1	11 February 2021	This patent described a method for the treatment of patients diagnosed with HER2-positive BC. The method involved the administration of a therapeutic amount of an antagonist of programmed cell-death (PCD) protein-1 combined with Trastuzumab and Pertuzumab [222].
21.	US 10,907,214B2	2 February 2021	Certain methods, devices, and kits to be used for the detection of biomarkers in cancer patients were disclosed in this patent. The treatment involved a secretory phospholipase A and a hydrolysable cisplatin-containing liposome. It also discussed the use of a microarray device with an oligonucleotidic probe to assess the responsiveness of the patient [223].
22.	US 2020/0385484 A1	10 December 2020	The compositions and procedures for treating cancer with chimeric antigen receptor-modified cells were included in the invention. An antigen-binding domain, a transmembrane domain, a co-stimulatory signalling region, and a CD3 zeta signalling domain may all be present in the antigen [224].
23.	US 10,858,626B2	8 December 2020	This invention provided methods for use in the adoptive immunotherapy of diagnosed cancer patients. It gave the composition, which consisted of specific tumour infiltrating lymphocytes, tyrosine-protein kinase-2 receptor, and a *vascular endothelial growth factor recepto* inhibitor [225].
24.	US 2020/0246275 A1	6 August 2020	This invention gave the methods for the treatment of an individual with cancer, which comprised the administration of a therapeutically effective amount of a nanoformulation of some taxanes with albumin and a Bcl-2 inhibitor such as ABT-263 [226].
25.	US 10,695,426B2	30 June 2020	This invention described a combination therapy consisting of antagonists of PCD-1 and lymphoma kinase for use in the treatment of cancer. The treatment consisted of monoclonal antibody with variable regions of seq. ID nos. 13 and 15 with a heavy chain and a light chain [227].
26.	US 2020/0157177 A1	21 May 2020	An immunotherapeutic strategy for cancer was discussed in this invention. It described the use of tumour-associated T-cell peptide epitopes in combination with certain other associated peptide molecules bound to the major histocompatibility complex [228].
27.	US 10,633,441B2	28 April 2020	This invention claimed that methods and compositions enhance the immune system response towards cancers. It related antigen receptors that selectively target human mesothelin [229].
28.	US 2020/0101102 A1	2 April 2020	This patent gave methods for immune response induction in cancer patients using Toll-like receptor-9 agonists being administered intratumourally [230].
29.	US 10, 596, 112B2	24 March 2020	This invention disclosed the composition and methods of making antibodies and carrier proteins to be used as cancer therapeutic agents. It also discussed a lyophilisation procedure for nanoparticle formulation that contained albumin, antibodies, and some drug such as Paclitaxel [231].
30.	US 10, 378, 066B2	13 August 2019	A molecular diagnostic test for cancer was presented in this invention. The test was based on the detection of DNA damage and the repair mechanism and was capable of determining cancer in clinically responsive or non-responsive patients [232].
31.	US 10, 370, 452B2	6 August 2019	This invention related to immunotherapeutics obtained from pluripotent stem cells to generate some phenotypical, functionally expandable T-cells. The cells could be used to target a specific antigen, thereby enhancing the cytotoxic potential, antitumour activity, and, thus, the survival of the patient [233].
32.	US 10, 344, 096B2	9 July 2019	This patent disclosed some pharmaceutical compositions and their preparation methods. The composition was said to be useful in inhibiting metastasis using receptor tyrosine kinase-like orphan receptor1 antibodies (ROR1-antibodies) [234].
33.	US 10, 266, 592B2	23 April 2019	This invention described surface-engineered human T-lymphocytes for augmenting the immune response. The designed molecules helped mediate cellular immunotherapy and, thus, were an effective strategy to treat cancer [235].
34.	US 10, 213, 499B2	26 February 2019	The use of proteins, peptides, and nucleic acids such as immunotherapeutic agents, either alone or in combination, was presented. The peptides could either be peptide epitopes or bound to molecules such as the major histocompatibility complex [236].
35.	US 10, 167, 290B2	1 January 2019	This invention reported substituted pyrazino [2,3-b] pyrazines as inhibitors of *mammalian target of rapamycin* (mTOR) kinase. These compounds are used for the treatment of diseases such as cancer, inflammation, or neurogenerative disease [237].
36.	US 10, 124, 023B2	13 November 2018	This patent explored the use of certain cells such as immune-responsive cells, natural killer cells, T-lymphocytes, and regulatory T-cells, which were capable of expressing an antigen-binding receptor in the activation of immune cells. These were single-chain variable fragments that bound to the antigens with immunosuppressive activity, thereby reducing the suppressive action of the antigen [238].
37.	US2018/0222997 A1	9 August 2018	Certain novel compositions and methods to diagnose and treat solid tumours by the use of antibodies were described. It was said that the antibody was specifically bound to the extracellular domain of the human Frizzled (FZD) *receptors,* thereby inhibiting growth of tumour cells [239].
38.	US 9, 920, 366B2	20 March 2018	This invention described the methods and systems for determining genetic variants and detecting double-stranded DNA [240].

## Data Availability

Not applicable.

## List of Abbreviations

AI	artificial intelligence
APCs	antigen-presenting cells
BC	breast cancer
BCV	breast cancer vaccine
CAD	computer aided diagnosis
CAV	carbohydrate antigen-based vaccine
CEA	carcino-embryonic antigen
CT	computed tomography
CTLA-4	cytotoxic T-lymphocyte antigen-4
CTLs	cytotoxic lymphocytes
DCIS	ductal carcinoma in situ
DCs	dendritic cells
DCV	dendritic cell-based vaccine
ER	estrogen receptor
FDGePET/CT	18F-fluorodeoxyglucose positron-emission tomography
FZD receptors	human Frizzled
GM-CSF	granulocyte-macrophage colony-stimulating factor
HER2	human epidermal growth receptor2
*h-TERT*	telomerase reverse transcriptase
ICB	immune checkpoint blockers
IDC	infiltrating ductal carcinoma
IFN-*γ*	interferon-*γ*
ILC	invasive lobular carcinoma
IORT	intraoperative radiation therapy
IT	immunotherapy
LCIS	lobular carcinoma in situ
MANPs	magnetic alloy nanoparticles
MDSCs	myeloid-derived suppressor cells
MHC	histocompatibility complex
MIAS	Mammographic Image Analysis Society
miRNAs	exosomal microRNAs
MMONPs	magnetic metal oxide nanoparticles
MNPs	magnetic nanoparticles
MRI	magnetic resonance imaging
MRM	multiple reaction monitoring
mTOR kinase	mammalian target of rapamycin kinase
*mtp53*	mutation in the *p*53 gene
MUC-1	mucin-1
MW	microwave
NK-cells	natural killer cells
NPs	nanoparticles
*p*53	tumour protein 53
PALP	placental alkaline phosphatase
PCD	programmed cell death
PCDL-1	programmed cell death ligand-1
PCDR-1	programmed cell death receptor-1
PET	positron emission tomography
PR	progesterone receptor
PRR	pattern recognition receptors
PV	peptide and protein-based vaccine
RFA	radiofrequency ablation
ROR1-antibodies	receptor tyrosine kinase-like orphan receptor1 antibodies
siRNAs	small interfering RNAs
STn	Sialy-Tn
TA	thermal ablation
TAMs	tumour-associated macrophages
TCR	T-cell receptor
TGF-*β*	transforming growth factor-*β*
TILs	tumour-infiltration lymphocytes
TLR	toll-like receptors
TME	tumour microenvironment
TNBC	triple-negative breast cancer
Treg cells	regulatory T-cells
TSA	tumour-specific antigens
WTCV	whole tumour cell-based vaccine
